# Supplementary material for: Aging Induces Profound Changes in sncRNA in Rat Sperm and These Changes Are Modified by Perinatal Exposure to Environmental Flame Retardant
Source: Int J Mol Sci. 2020 Nov 4;21(21):8252. doi: 10.3390/ijms21218252 (PMC7672616; doi:10.3390/ijms21218252)
Supplement: Supplementary file 1 [file ijms-21-08252-s001.zip › Supplemental figures 1-5.pptx]

## Slide 1
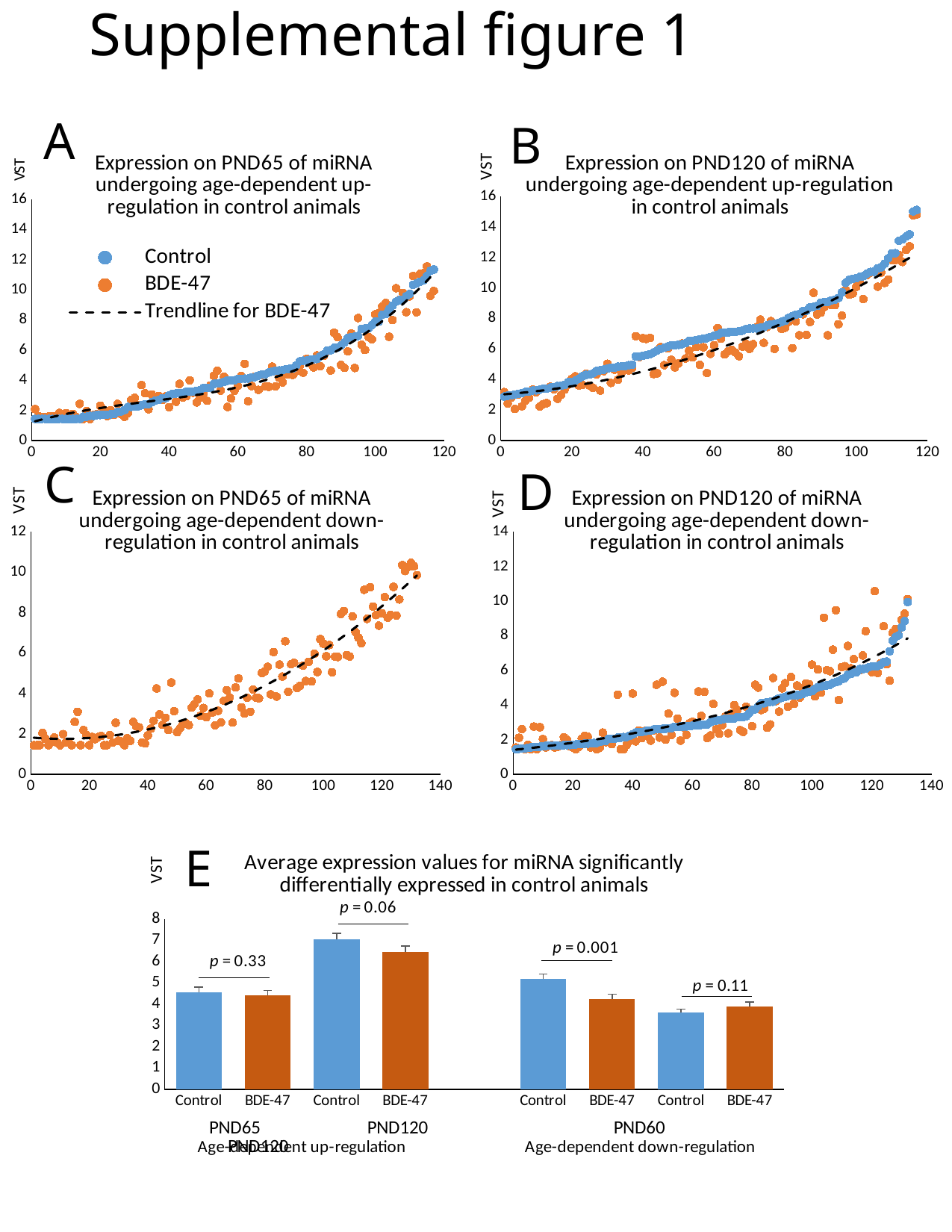

Supplemental figure 1
A
B
### Chart: Expression on PND65 of miRNA undergoing age-dependent up-regulation in control animals
| Category | | |
|---|---|---|
### Chart: Expression on PND120 of miRNA undergoing age-dependent up-regulation in control animals
| Category | Control PND120 | BDE-47 PND120 |
|---|---|---|C
D
### Chart: Expression on PND65 of miRNA undergoing age-dependent down-regulation in control animals
| Category | 2.151539982 | 1.432378289 |
|---|---|---|
### Chart: Expression on PND120 of miRNA undergoing age-dependent down-regulation in control animals
| Category | Mean C120 | Mean E120 |
|---|---|---|E
### Chart: Average expression values for miRNA significantly differentially expressed in control animals
| Category | |
|---|---|
| Control | 4.553418018188272 |
| BDE-47 | 4.399202504682983 |
| Control | 7.04917706888741 |
| BDE-47 | 6.453646020907665 |
| | None |
| Control | 5.199963166158827 |
| BDE-47 | 4.251396680473874 |
| Control | 3.5959758729166893 |
| BDE-47 | 3.9021532820634754 |p = 0.11

## Slide 2
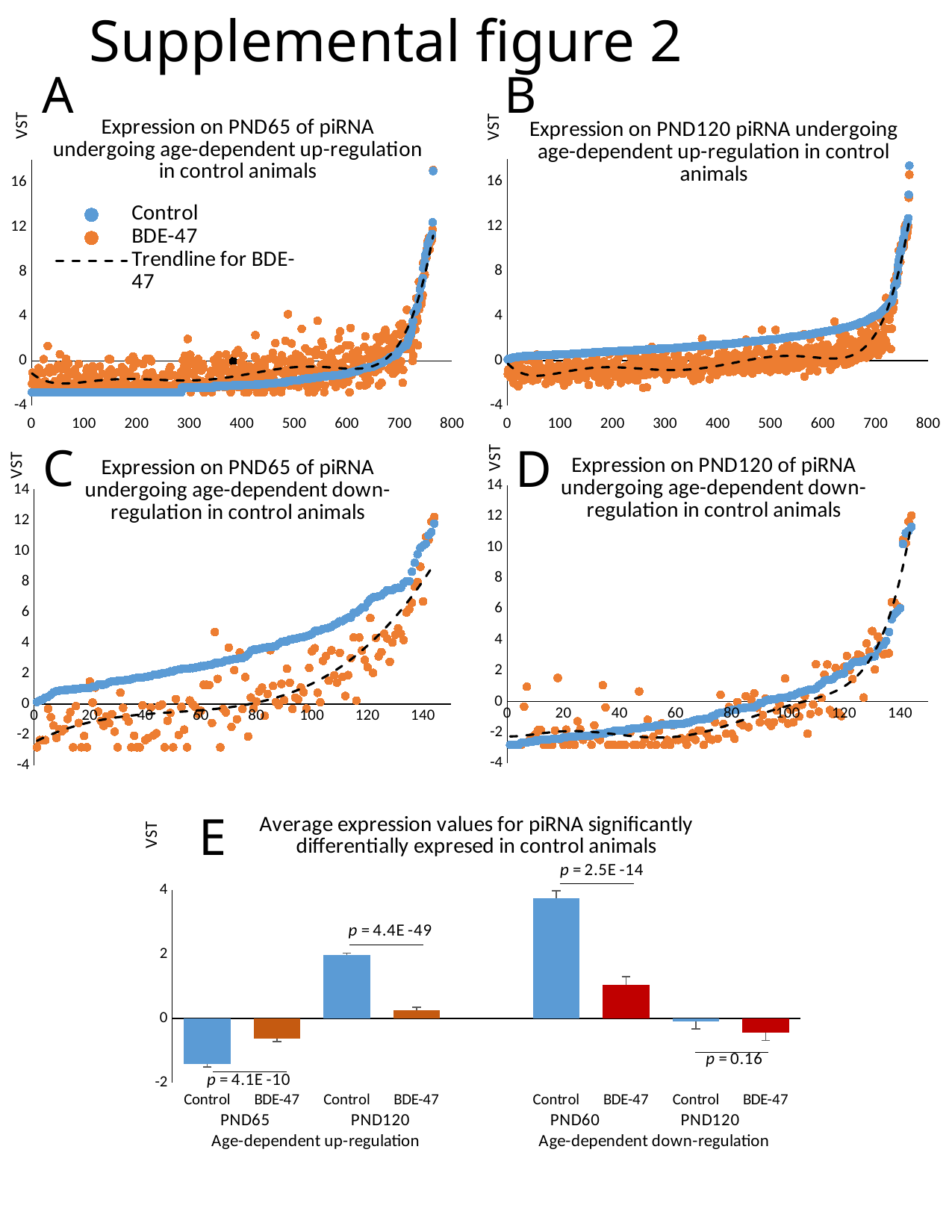

Supplemental figure 2
A
B
### Chart: Expression on PND65 of piRNA undergoing age-dependent up-regulation in control animals
| Category | | |
|---|---|---|
### Chart: Expression on PND120 piRNA undergoing age-dependent up-regulation in control animals
| Category | | |
|---|---|---|C
### Chart: Expression on PND120 of piRNA undergoing age-dependent down-regulation in control animals
| Category | Control | BDE-47 |
|---|---|---|D
### Chart: Expression on PND65 of piRNA undergoing age-dependent down-regulation in control animals
| Category | Control | BDE-47 |
|---|---|---|
### Chart: Average expression values for piRNA significantly differentially expresed in control animals
| Category | |
|---|---|
| Control | -1.427477454032435 |
| BDE-47 | -0.6426681014619092 |
| Control | 1.9693606069456877 |
| BDE-47 | 0.26091532430437625 |
| | None |
| Control | 3.763624486139867 |
| BDE-47 | 1.0478576846204817 |
| Control | -0.10349152172177564 |
| BDE-47 | -0.4441973148949624 |E

## Slide 3
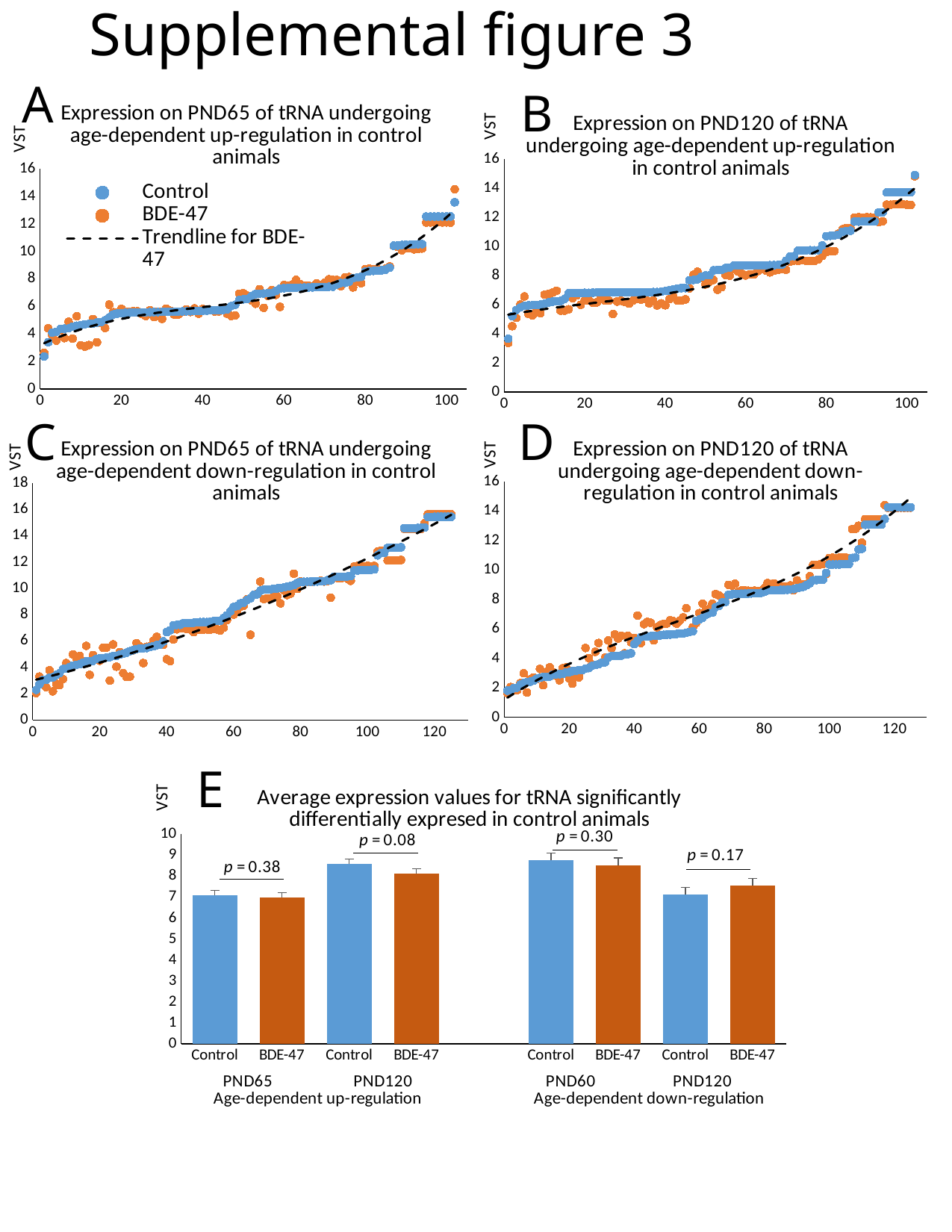

Supplemental figure 3
A
B
### Chart: Expression on PND65 of tRNA undergoing age-dependent up-regulation in control animals
| Category | | |
|---|---|---|
### Chart: Expression on PND120 of tRNA undergoing age-dependent up-regulation in control animals
| Category | Control PND120 | BDE-47 PND120 |
|---|---|---|C
D
### Chart: Expression on PND65 of tRNA undergoing age-dependent down-regulation in control animals
| Category | | |
|---|---|---|
### Chart: Expression on PND120 of tRNA undergoing age-dependent down-regulation in control animals
| Category | | |
|---|---|---|E
### Chart: Average expression values for tRNA significantly differentially expresed in control animals
| Category | |
|---|---|
| Control | 7.076378586244031 |
| BDE-47 | 6.979665077564908 |
| Control | 8.577175908727016 |
| BDE-47 | 8.1158694101629 |
| | None |
| Control | 8.769288202158181 |
| BDE-47 | 8.522196732891933 |
| Control | 7.135440821833801 |
| BDE-47 | 7.560381678046385 |

## Slide 4
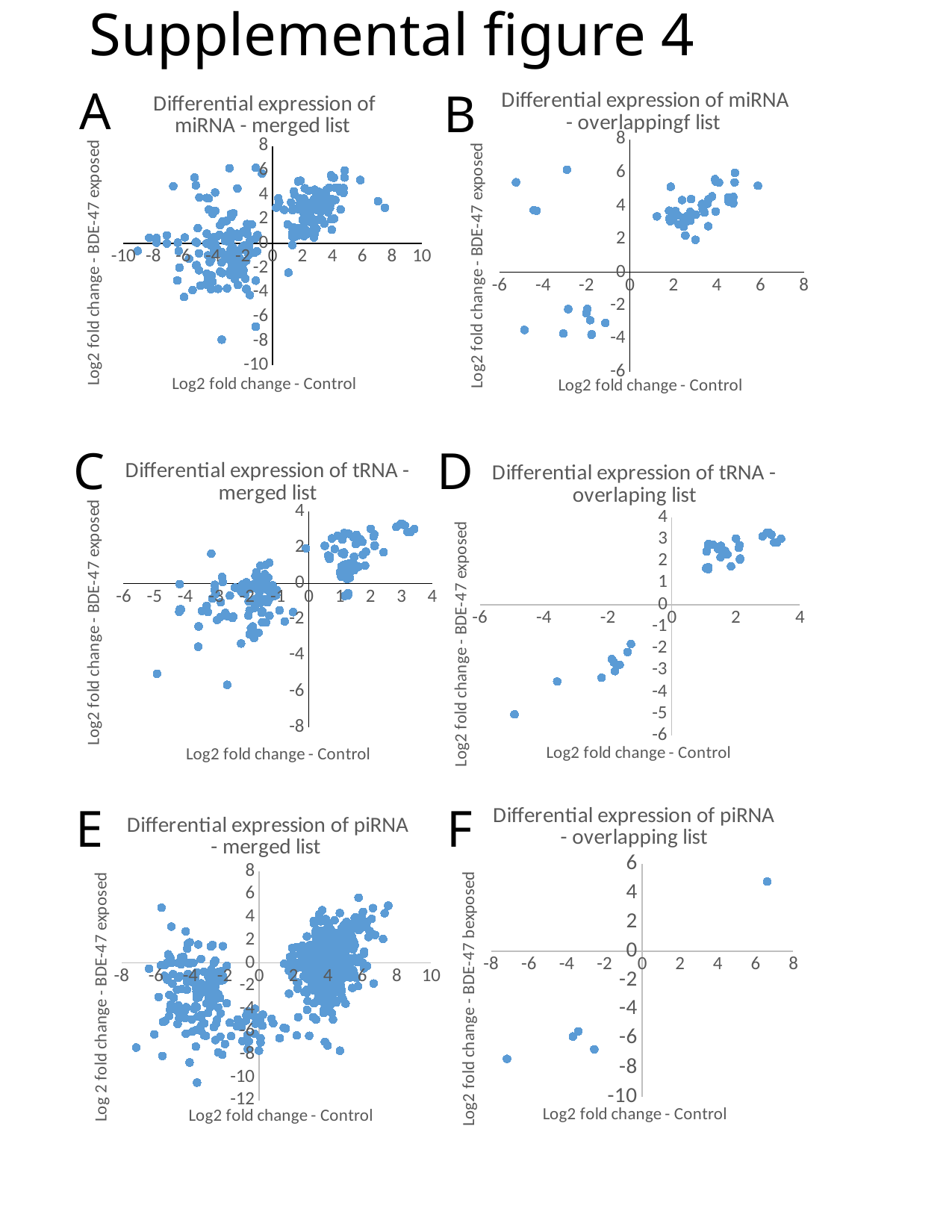

Supplemental figure 4
### Chart: Differential expression of miRNA - overlappingf list
| Category | Eposed Log2FCh |
|---|---|
### Chart: Differential expression of miRNA - merged list
| Category | Eposed Log2FCh |
|---|---|A
B
C
D
### Chart: Differential expression of tRNA - merged list
| Category | Exposed Log2FCh |
|---|---|
### Chart: Differential expression of tRNA - overlaping list
| Category | Exposed Log2FCh |
|---|---|F
E
### Chart: Differential expression of piRNA - overlapping list
| Category | |
|---|---|
### Chart: Differential expression of piRNA - merged list
| Category | |
|---|---|

## Slide 5
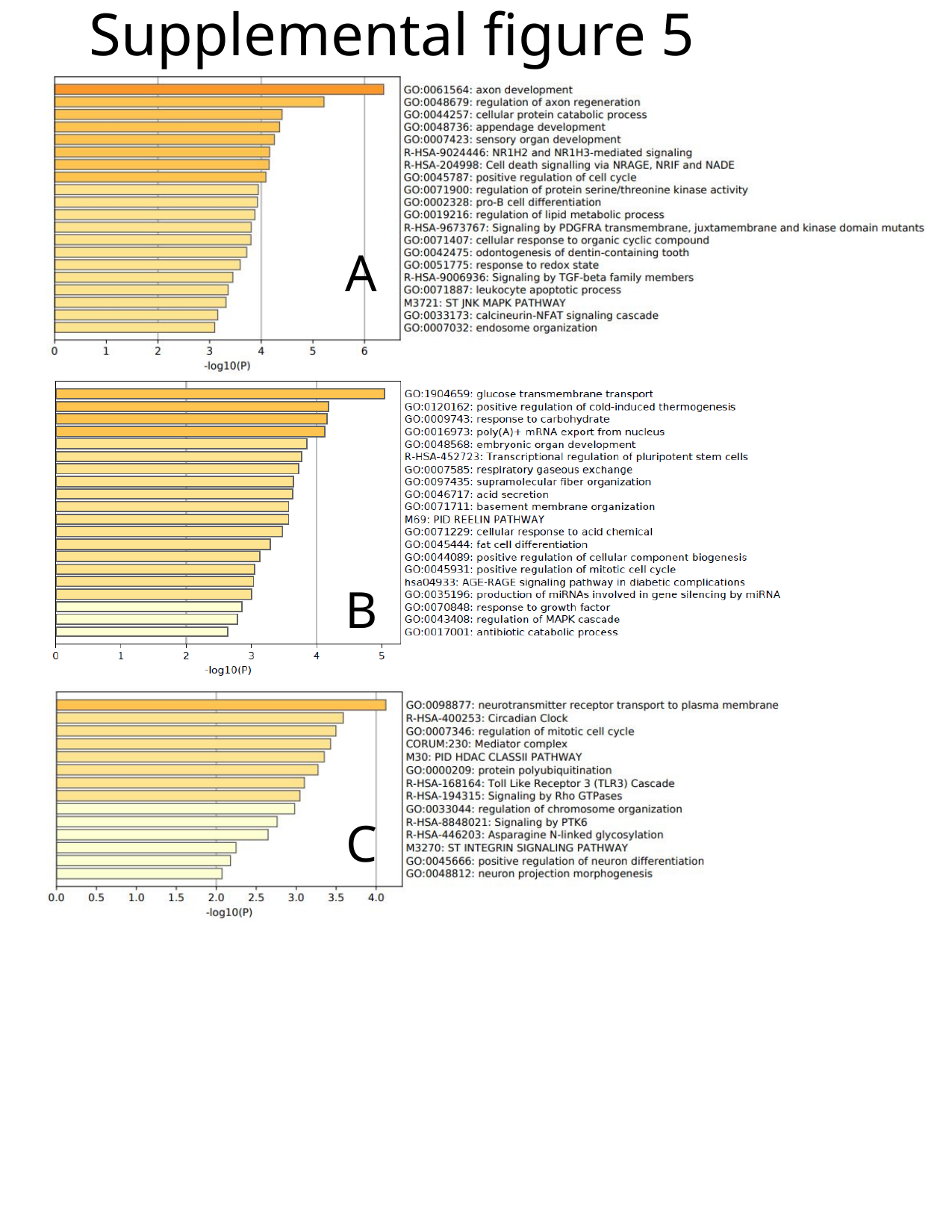

Supplemental figure 5
A
B
C
